# Acculturation and diet quality of young Filipino children in the United States Affiliated Pacific Islands

**DOI:** 10.21203/rs.3.rs-8961066/v1

**Published:** 2026-03-24

**Authors:** Monica Esquivel, Ashley Yamanaka, Sabine Strasburger, Patricia Coleman, Tanisha Aflague, Kristi Hammond, Lynne Wilkens, Rachel Novotny

**Affiliations:** University of Hawai‘i at Mānoa; University of Hawai‘i at Mānoa; University of Hawai‘i at Mānoa; Northern Marianas College; University of Guam; University of Hawai‘i at Mānoa; University of Hawai‘i Cancer Center; University of Hawai‘i at Mānoa

## Abstract

Filipinos are among the largest ethnic groups in Hawai‘i, Guam, and the Commonwealth of the Northern Mariana Islands (CNMI), yet little is known about the nutritional status of Filipino children in these jurisdictions which is critical to disease prevention among Filipino adults. Nutritional status and intake of foods and nutrients of Filipino children from Hawai‘i, Guam, and CNMI was compared using cross-sectional analysis of data from the Children’s Healthy Living (CHL) Program prevalence study (2013–2014). The sample included 446 Filipino children in Hawai‘i, Guam, and CNMI. Demographics, weight and height status, Healthy Eating Index (HEI)-2020 scores, and nutrient intakes were measured across the three jurisdictions. Survey-weighted models compared demographics, weight status, diet quality, and nutrient intakes across jurisdictions. Mean HEI-2020 scores were suboptimal (50.1 ±0.99), with the highest in CNMI (52.1), followed by Hawai‘i (51.1), and Guam (48.9). White rice and two-percent milk were dominant contributors to multiple nutrients in all jurisdictions. Young Filipino children in the Pacific can improve diet quality. Variation in diet intake across jurisdictions call for culturally and geographically tailored nutrition interventions.

## Background

Filipinos are the second largest ethnic group in the state of Hawai‘i^1^ and Guam^2^ and the largest ethnic group in the Commonwealth of the Northern Mariana Islands (CNMI)^3^. One in four Hawai‘i residents are of Filipino ancestry and one in three in both Guam and CNMI. Filipino adults experience higher rates of chronic conditions such as hypertension^4^, diabetes^5^, obesity^6^, and asthma^7^; while Filipino children living in the Philippines experience a triple burden of malnutrition, undernutrition (wasting and stunting), micronutrient deficiencies, and overnutrition (overweight and obesity).^8–10^ In the US, these health disparities are often masked as data on Filipino populations are often aggregated within the Asian category.^11,12^ Furthermore, little is known about the nutritional or health status of Filipino children living outside of the Philippines, which is key to identifying strategies to prevent chronic disease development in adults. Of the limited studies, one study in California identified that Filipino children had higher odds of several obesogenic dietary practices in comparison to Chinese children; this included low vegetable intake, high sugar sweetened beverage intake, and high fruit juice intake.^13^ In another small study of Filipino children and caregivers (n=28) in Hawai‘i, differences in eating patterns among ethnic groups were examined.^14^ Filipino children (n=28) had lower dairy intake than White children (n=18). All children had low intakes of fruits, vegetables, and whole grains compared to recommendations for children, while added sugar intakes were high.^14^

Changes in the dietary intakes of Filipinos can be explained by both the nutrition transition and dietary acculturation. Where the nutrition transition, a global phenomenon, refers to the shift from traditional to western diets which are high in ultra-processed foods,^15^ and dietary acculturation, which occurs with migration, refers to the process by which a minority population adopts food choices and eating patterns of the host country.^16^ Both are contributors to the shifting dietary habits and subsequent health disparities observed in this population. Western acculturation among Filipino adults has been associated with increased calorie intake, percent of fat intake, BMI and waist circumference.^17,18^ The migration of Filipinos to CNMI, Guam, and Hawai‘i has occurred over different waves, where in CNMI migration occurred primarily after World War II^19^, in Guam it began with the Spanish occupation of the Philippines and Guam during the late 19th century^19^, and in Hawai‘i during the early 1900s^20^, Therefore, understanding differences in recency of migration and acculturation among Filipinos living in these different communities and the impact on diets of children is needed. The objective of this research is to compare the nutritional status and dietary intake of foods and nutrients of Filipino children from across Hawai‘i, Guam, and CNMI and explore differences in acculturation and years of residence in each jurisdiction.

## Methods

Data were drawn from the Children’s Healthy Living (CHL) Program, a prevalence study and intervention trial conducted from 2013 – 2015 across 11 jurisdictions in the United States Affiliated Pacific region, described elsewhere.^21^ Briefly, the CHL Program collected comprehensive health and dietary data from children ages two to eight years of age from 2013–2014. The cross-sectional data included demographic data, two-day food and activity records, and anthropometric measurements that were collected by trained staff in 24 communities that met selection criteria of having at least 25% indigenous populations.^21^ A total of 4178 children ages 2–8 years participated in the prevalence study, of which 3529 provided two nonconsecutive days of food records. Parents provided written informed consent and children provided assent to participate and receive compensation prior to participation. Compensation level, determined by jurisdiction lead investigators and their IRB’s, was higher in Guam (University of Guam, $40) than in Hawai‘i (University of Hawai‘i at Mānoa, $20). CNMI ceded IRB approval and compensation rate to the University of Hawai‘i at Mānoa. The analytic sample for this study included 446 children of Filipino ancestry with dietary records residing in Hawai‘i, Guam, and CNMI. Filipino ancestry was identified from caregiver-reported ethnicity.

### Demographics

Caregivers reported household income, child’s age, sex, and race or ethnicity which included subcategories in addition to the Office of Management and Budget^22^ categories: under Asian, Native Hawai‘ian and Pacific Islanders, and American Indian/Alaska Native, including a write-in opportunity. Filipino was a subcategory included under Asian and was used to determine inclusion in this study, which includes any-part Filipino. Questions were also included on the demographic form related to language spoken at home, place of birth, the number of years the child lived in the jurisdiction, and cultural affiliation which measured knowledge, involvement, beliefs and association with traditional group culture and lifestyle.^23^

### Acculturation

Acculturation scores were determined through an adapted questionnaire to allow for the evaluation of acculturation/enculturation.^24^ Acculturation questions were divided into 2 subscales: a 4-item ethnic traditional cultural identity subscale and a 4-item US mainland cultural identity subscale. These questions assessed for the traditional and US mainland culture, the personal feelings, knowledge of practices, association with others, and impact the culture had on their lifestyle. For each domain a five-point subscale was used from one (very knowledgeable; very involved; very positive; most of the time) to five (not at all knowledgeable; not at all involved; very negative; not at all associated). Total scores were summed from each subscale and categorized into one of four acculturation categories: 1) integrated (high affiliation with traditional and US mainland identities), 2) traditional (high affiliation with traditional identity only), 3) assimilated (high affiliation with US mainland identity only) and 4) marginalized (low affiliation with ethnic and US mainland identity).^21^ Due to the small sample, assimilated and marginalized categories were combined.

### Anthropometrics

Trained CHL staff measured weight (in kilograms), height (in centimeters), and waist circumference (in centimeters) using a standardized protocol.^25^ The mean of three measures was used. Body mass index (BMI)-for-age z-scores were determined by the Centers for Disease Control and Prevention (CDC) growth charts,^26^ categorized as underweight (<5th percentile), healthy weight (5th to the 84th percentile), overweight (85th and 94th percentile) and obese (≥95th percentile). Stunting was defined as current height-for-age >2 SDs below the mean of the CDC reference mean.^27^

### Dietary Assessment

Caregivers recorded all foods and beverages their child consumed (descriptions and quantities), for two systematically assigned, non-consecutive days of the week, which included both weekdays and weekend days. Additional instructions were provided to parents/caregivers on reporting dietary intake of children away from home (i.e., at school or child care settings). Completed food records were returned to the researchers one week later and with the parent/caregiver present, the researchers reviewed records, probed parents/caregivers for completeness, added details, and made corrections as needed.^23^Dietary supplements were not included. Food records were reviewed by staff for completeness and entered into Pacific Tracker 3 (PacTrac3), a comprehensive nutrient database and web application that includes information on unique foods and recipes from the Pacific Islands.^28^ PacTrac3 provides an accurate estimation of food and nutrient intake based on United States Dietary Guidelines and the Healthy Eating index. After entry into PacTrac, trained researchers reviewed dietary records with extreme nutrients or food responses to correct errors; thus no records were removed, to avoid losing dietary variability.^29^

Food and dietary components were averaged across days, adjusting for weekday and weekend days and corrected for within-person variance to minimize bias using the Statistical Program to Assess Dietary Exposure package in R (R Core Team, Vienna, Austria, 2013). After de-attenuation and averaging, all records fell in a reasonable range for energy and other dietary components, as defined by extreme values: energy ≥3755 kcal or ≤500 kcal, fruit intake ≥ 5 cups, vegetable intake ≥ 3 cups, vitamin A intake ≥ 2005 ug, and water ≥5 cups. When extreme values were identified, entries were checked for missing information, serving sizes, and quantities to ensure they were entered correctly. Diet data analysis accounted for the complex survey design, including the clustering of children in communities within jurisdiction strata, and the weighting to reflect the population size of children across the three jurisdictions. The data were used to calculate intakes of food groups: fruits; vegetables; grains (both refined and whole grains); milk; and meat; and intakes of energy and nutrients.^28,30^ The US MyPlate food guidance system was used to group foods and beverages reported into food groups and to assign cup or ounce equivalents.^28,31^

The top five food contributors to the food groups, macronutrient, and micronutrient intake among consumed food items were identified based on a percentage of the nutrients derived from that specific food item in the population.

The Healthy Eating Index (HEI)-2020 is an updated version of the diet quality index developed by USDA which compares diets to US dietary guidance.^32^ The HEI-2020 is composed of 13 dietary components with a standard minimum (0) and maximum (5 or 10) score for each dietary component and a total score ranging between 0–100 points. Nine adequacy components include Total Fruits, Whole Fruits, Total Vegetables, Greens and Beans, Whole Grains, Dairy, Total Protein Foods, Seafood and Plant Proteins, and Fatty Acids. Four moderation components include Refined Grains, Sodium, Added Sugars, and Saturated Fats, reverse-scored where a higher score is equivalent to lower intake. Most components are scored on a density basis, i.e. amounts per 1,000 kcal of total energy intake, with the exception of Fatty Acids, which is scored as a ratio of poly- and monounsaturated fatty acids (PUFAs and MUFAs) to saturated fatty acids (SFAs), and Added Sugars and Saturated Fats, which are scored as the percentage of total energy intake.

### Statistical analysis

Descriptive statistics for demographic variables included frequencies and percentages for the total sample and by jurisdiction group. Comparisons of socio-demographic characteristics such as age, sex, household income, acculturation, languages spoken at home, weight status (BMI-for-age categories^26^) of Filipino children between jurisdictions were analyzed using chi square and analysis of variance. Prevalence and means, with standard error, were estimated using survey sampling techniques that weighted the sample to the young child population size in each community based on census data and accounted for the clustering of participants in communities within jurisdictions. The weighting resulted in representative estimates for the jurisdictions and the region. Hierarchical marginal linear models of food groups, macronutrients, micronutrients, and HEI-2020 scores (adjusting for age, sex, and dietary energy) were fit to compare means between subgroups. Distributions of residuals were checked to ensure that model assumptions were met; no dietary variable required transformation. Dietary variables were analyzed and compared among jurisdictions. A p-value <0.05 was considered statistically significant. Statistical analysis was done using SAS 9.4 (SAS Institute Inc).

## Results

Among the 446 Filipino children, 43.5% resided in CNMI (n = 194), 24.9% (n = 111) in Guam, and 31.6% (n = 141) in Hawai‘i (Table 1). Children were primarily between 2–5 years old (62.6%), though age distribution varied by jurisdiction. In Hawai‘i, nearly four out of five children (78.0%) were two to five years old, compared with 60.8% in CNMI and 46.0% in Guam. Sex distribution did not differ across jurisdictions.

Most children lived in households with annual incomes below $35,000 (77.4%). This proportion was highest in CNMI (96.2%), compared with 63.4% in Guam and 63.6% in Hawai‘i. The average time children had lived in their jurisdictions was 5.2 years (SE = 0.1), ranging from 4.6 years in Hawai‘i to 5.6 years in Guam. English was the predominant language spoken at home (89.4%), with a Filipino dialect only (2.2%) or English plus a Filipino dialect and/or another language (8.4%) reported less frequently. Language use differed across jurisdictions with 97.9% of children in Hawai‘i speaking English only, and 14.6% of children in CNMI speaking both English and a Filipino dialect and/or another language. Acculturation scores indicated that most children were classified as integrated (81.0%), followed by traditional (14.6%), and assimilated or marginalized (4.4%). Traditional orientation was most common in Hawai‘i (20.4%), while Guam had the lowest proportion of traditional classification (6.5%).

Distribution across the BMI categories was 5% underweight, 68% healthy weight, 12% overweight, and 16% obese, with no jurisdictional differences (Table 1). The prevalence of obesity ranged from 14.2% in Hawai‘i to 16.8% in Guam and 16.1% in CNMI. Stunting prevalence was highest among children in CNMI (7.6%) and lowest in Hawai‘i (2.1%).

**Figure 1:**
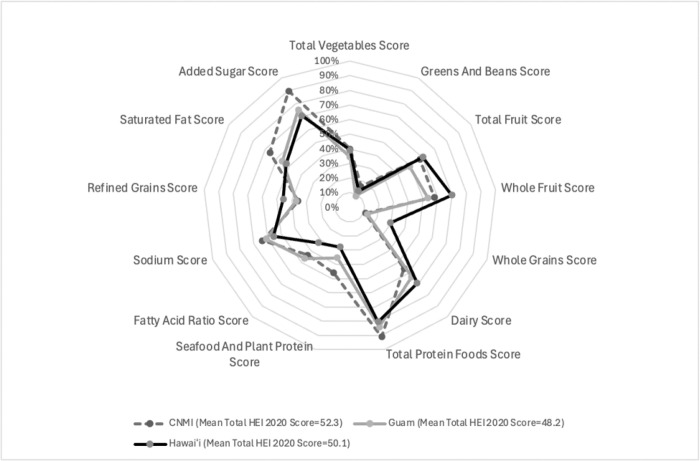
Adjusted mean Healthy Eating Index-2020 (HEI 2020) component scores for two to eight year old Filipino children enrolled in the Children’s Healthy Living Program (2012–2014), by jurisdiction. Sodium, Refined Grains, Saturated Fat, and Added Sugar are moderation components. Models were adjusted for age, sex, and energy intake and accounted for complex survey design.

Overall, diet quality was low with a mean HEI-2020 total score of 50.67 (SE = 0.99), below US national averages for children (Table 2). Scores differed by jurisdiction (p = 0.002): children in CNMI had the highest score (52.33, SE = 0.47), followed by Hawai‘i (50.12, SE = 2.17) and Guam (48.16, SE = 0.69). Notable HEI component score differences included that Hawai‘i children scored higher on whole fruit (p = 0.04), whole grain (p = 0.003), dairy (p = 0.04), and refined grain scores (p = 0.09). Children from CNMI scored higher on seafood and plant protein (p<.0001) and added sugar (p = < .0001). Guam children consistently scored lowest across most components (Fig. 1)

Food group consumption differed across jurisdictions in ways that mirrored diet quality scores. Hawai‘i children consumed the most dairy (1.55 cups, SE = 0.14), while CNMI children consumed the most meat (6.26 oz, SE = 0.19). Guam children had lower fiber (8.64 g, SE = 0.24) intake compared to both CNMI and Hawai‘i.

Nutrient intake differences were evident for protein, fiber, fatty acids, and multiple vitamins and minerals. For example, calcium intake was highest in Hawai‘i (763.27 mg, SE = 30.82), while selenium intake was highest in CNMI (106.07 mg, SE = 0.78). Children in Guam had lower intakes of several micronutrients, including choline (282.41 mg, SE = 4.95) and vitamin B6 (1.58 mg, SE = 0.08). Table 3 contains a full list of nutrient and food intake for the total group and by jurisdiction.

Across all jurisdictions, Filipino children’s diets were dominated by a small set of staple foods. White rice was the leading source of energy, carbohydrates, protein, and several micronutrients, while two-percent milk was the top source of calcium, phosphorus, and vitamins A, B12, and D (Table S1). Together, rice and milk accounted for a substantial share of total nutrient intake.

Sugar-sweetened beverages also played a notable role. Sweetened iced tea was the largest contributor of added sugars in all jurisdictions (Table S2). Other commonly consumed foods included white bread, eggs, chicken, and processed meats such as sausage and spam. While these foods provided key nutrients, they also contributed to high saturated fat, sodium intake, and nitrites (Table S3).

## Discussion

Our findings show clear jurisdictional differences among Filipino children in socioeconomic status, acculturation, and dietary intake, with CNMI children having the highest diet quality scores and Guam the lowest. Dietary differences were particularly evident in meat, whole grain, dairy, and added sugar consumption. Across jurisdictions, white rice and 2% milk were the predominant dietary sources of multiple nutrients, underscoring both the cultural centrality of rice and the influence of Westernized food environments. These findings highlight the importance of considering geographic differences when developing nutrition interventions for Filipino children in the Pacific region.

Despite differences in household income and language use, BMI distribution and obesity prevalence were similar across jurisdictions. The 15.7% prevalence of obesity in this Filipino child subsample is comparable to national estimates for Filipino children in urban areas of the Philippines (15.8%)^9,33,34^ but slightly higher than the overall CHL cohort at baseline (14%).^27^ The highest prevalence of underweight was in the CNMI, consistent with the greater proportion of low-income households there, and echoing evidence from the Philippines that undernutrition disproportionately affects children in lower-income households.^8^

The apparent disconnect between diet quality scores and obesity prevalence across jurisdictions warrants further explanation. While CNMI children had the highest HEI-2020 scores, obesity prevalence was similar across all three locations. This suggests that factors beyond overall diet quality, such as total energy intake, physical activity patterns, or specific dietary components not captured by HEI-2020, may be driving obesity risk. The dominance of energy-dense staples like white rice across all jurisdictions, regardless of other dietary differences, may explain this pattern. Additionally, the HEI-2020 may not fully capture the health implications of food processing levels or cultural food preparation methods that could influence obesity.

In this subsample of Filipino children, the mean HEI score was 50.67, with Guam exhibiting the lowest mean score (48.16) and the CNMI the highest (52.33). These values were consistently lower than those reported in a prior study examining HEI scores in the 2025 US Dietary Guidelines for Americans, for which diet quality ranged from 53 to 59 (based on age and sex), but similar to the diet quality scores of the broader CHL cohort.^35,36^ Notably, intake of whole grains in this sample of Filipino children were slightly lower than the broader CHL cohort (1.7 vs 2.1).^36^

White rice was the dominant contributor to energy, protein, carbohydrates, and several B vitamins and minerals, while 2% milk was a major source of calcium, phosphorus, and vitamins A, B12, and D. These findings emphasize the nutritional double-edged sword of staple foods and are consistent with recent findings from the 2023 Philippines National Nutrition Survey, where rice made up 50% of total energy intake and was the top contributor of dietary protein.^37,38^ While white rice and milk provide essential nutrients, they may also displace more nutrient-dense foods and contribute to high saturated fat and refined carbohydrate intake. The reliance on sweetened beverages, particularly iced tea, as the leading source of added sugars is a concern.

Differences in dietary intake among jurisdictions may reflect variation in migration history, food availability, and acculturation, though our findings suggest that local food environments may be equally important drivers of dietary patterns. While migration history likely influences some food choices, all jurisdictions showed heavy reliance on processed foods and sugar-sweetened beverages, indicating that Westernization of food systems affects dietary patterns regardless of individual acculturation status. Filipino children in CNMI represent a more recent wave of migration compared to Hawai‘i, which may explain higher consumption of traditional foods alongside economic constraints. Conversely, in Hawai‘i, longer-term settlement and greater food access may have shaped diets toward greater inclusion of dairy and packaged foods. As Filipino families migrate to places like Guam, CNMI, or Hawai‘i, their traditional diets often shift towards more Westernized eating patterns.^12,34^ However, differences in food availability, access, and cost can impact the types of foods consumed in these locations. These findings suggest that acculturation interacts with local food environments, shaping dietary practices differently across Pacific communities.

These findings suggest the need for nutrition interventions tailored to both cultural food practices and local food environments. Given that sweetened iced tea was the primary source of added sugars across all jurisdictions, targeted interventions around beverage consumptions could yield significant improvements. This might include beverage policies in schools, community education campaigns, or working with local retailers to promote unsweetened alternatives. The heavy reliance on white rice presents both a challenge and opportunity. While a complete substitution of white rice may not be culturally acceptable or economically feasible, interventions could focus on preparation methods, portion control, or partial substitutions with high-fiber alternatives like brown rice or starchy tubers (sweet potatoes). Interventions could focus on: encouraging whole grain substitutions for refined rice products, promoting fruits and vegetables through culturally relevant approaches, reducing intake of sugar-sweetened beverages, and leveraging existing dietary staples (eg. rice and milk) as vehicles for fortification or healthier preparation practices. Given the high proportion of Filipino people in USAP jurisdictions, targeted strategies are essential for addressing disparities in chronic disease risk across the life course.

Several limitations should be noted. First, dietary intake was based on two-day food records, which may not fully capture usual intake patterns, particularly for foods consumed less frequently or seasonally. This limitation may be relevant for traditional Filipino foods that might be prepared for special occasions. Second, Filipino children were sampled within communities selected for high Pacific indigenous representation, which may limit generalizability to urban Filipino populations or those in communities with different demographic compositions. Third, the acculturation scale used was not specific to Filipino populations and may not capture culturally relevant aspects of dietary change specific to this community. Despite these limitations, this study provides novel insights into the diets of Filipino children in the Pacific.

## Conclusion

Young Filipino children from the CHL sample in Hawai‘i, Guam, and CNMI exhibit suboptimal diet quality with heavy reliance on refined rice, milk, and sugar-sweetened beverages. Jurisdictional differences reflect socioeconomic variation, migration history, and food environments. These findings highlight the importance of culturally and geographically tailored nutrition policies and programs to improve diet quality and reduce chronic disease risk in Pacific Island Filipino populations.

## Supplementary Material

This is a list of supplementary files associated with this preprint. Click to download.

• CHLFilpinoDietPaperforHCSupplementaryTables.docx

## Figures and Tables

**Table 1:** Description of Filipino children by Pacific jurisdiction, the Children's Healthy Living Program.

Characteristics	Total (n = 446)	CNMI^a^ (n = 194)	Guam (n = 111)	Hawaiʻi (n = 141)	P-value^b^
**Age Group, n (%)**					
2–5 years	279 (62.6)	118 (60.8)	51 (46.0)	110 (78.0)	<.0001
6–8 years	167 (37.4)	76 (39.2)	60 (54.0)	31 (22.0)	
**Sex, n (%)**					
Male	237 (53.1)	109 (56.2)	59 (53.2)	69 (48.9)	0.2116
Female	209 (46.9)	85 (43.8)	52 (46.8)	72 (51.1)	
**Household income, n (%)**					
less than $35,000	287 (77.4)	151 (96.2)	59 (63.4)	77 (63.6)	<.0001
$35,000 or greater	84 (22.6)	6 (3.8)	34 (36.6)	44 (36.4)	
**Years in jurisdiction, mean (SE^c^)**	5.2 (0.1)	5.5 (0.1)	5.6 (0.2)	4.6 (0.2)	<.0001
**Languages Spoken at home n (%)**					
English Only	397 (89.4)	158 (82.3)	101 (91.0)	138 (97.9)	<.0001
Filipino Only	10 (2.2)	6 (3.1)	4 (3.5)	0 (0)	
English and Filipino and/or Other Language	37 (8.4)	28 (14.6)	6 (5.5)	0 (0)	
**Acculturation Score**^23^ **n (%)**					
Integrated	349 (81.0)	152 (81.7)	96 (88.9)	101 (73.7)	0.0258
Traditional	63 (14.6)	28 (15.1)	7 (6.5)	28 (20.4)	
Assimilated/Marginalized	19 (4.4)	6 (3.2)	5 (4.6)	8 (5.9)	
**BMI Category n (%)**					
Underweight	21 (4.8)	12 (6.2)	6 (5.6)	3 (2.1)	0.1994
Healthy weight	299 (67.8)	130 (67.4)	71 (66.4)	98 (69.5)	
Overweight	52 (11.8)	20 (10.4)	12 (11.2)	20 (14.3)	
Obese	69 (15.7)	31 (16.1)	18 (16.8)	20 (14.2)	
**Stunting n (%)**					
No	419 (95.9)	183 (96.3)	98 (92.5)	138 (97.9)	0.0972
Yes	18 (4.1)	7 (3.7)	8 (7.6)	3 (2.1)	

a CNMI= Commonwealth of the Northern Mariana Islands.

b P-value comparing jurisdictions using the chi-square test for categorical variables and ANOVA for continuous variables.

c SE=Standard Error

**Table 2:** Healthy Eating Index (HEI) 2020 Component of Filipino Children by Pacific Jurisdiction, adjusted for age, sex, and energy intake.

Dietary Intake	Total	CNMI^a^	Guam	Hawai⊠i	P-value
***HEI 2020 Component Scores (range)***					
Total Vegetables Score (0 to 5)	1.91 (0.22)	2.00 (0.20)	1.72 (0.20)	1.96 (0.20)	0.20
Greens And Beans Score (0 to 5)	0.67 (0.06)	0.81 (0.07)	0.44 (0.06)	0.64 (0.11)	**0.01**
Total Fruit Score (0 to 5)	2.84 (0.16)	2.9 (0.14)	2.47 (0.18)	3.02 (0.41)	0.19
Whole Fruit Score (0 to 5)	3.03 (0.14)	2.9 (0.17)	2.68 (0.05)	3.50 (0.29)	**0.04**
Whole Grains Score (0 to 10)	1.73 (0.28)	1.13 (0.09)	1.26 (0.17)	2.94 (0.35)	**0.003**
Dairy Score (0 to 10)	6.15 (0.25)	5.56 (0.27)	6.34 (0.24)	6.91 (0.35)	**0.04**
Total Protein Foods Score (0 to 5)	4.32 (0.11)	4.55 (0.07)	4.20 (0.06)	4.02 (0.25)	**0.001**
Seafood And Plant Protein Score (0 to 5)	1.88 (0.15)	2.30 (0.05)	1.77 (0.03)	1.39 (0.19)	**< .0001**
Fatty Acid Ratio Score (0 to 10)	4.13 (0.21)	4.34 (0.14)	4.65 (0.12)	3.23 (0.08)	**< .0001**
Sodium Score (0 to 10)	6.07 (0.27)	6.38 (0.35)	6.10 (0.28)	5.57 (0.46)	0.40
Refined Grains Score (0 to 10)	3.89 (0.19)	3.55 (0.11)	3.69 (0.16)	4.58 (0.41)	0.09
Saturated Fat Score (0 to 10)	5.97 (0.25)	6.61 (0.07)	5.59 (0.21)	5.27 (0.20)	**0.0003**
Added Sugar Score (0 to 10)	8.07 (0.34)	8.97 (0.10)	7.54 (0.07)	7.07 (0.13)	**< .0001**
HEI-2020 Total Score (o to 100)	50.67 (0.99)	52.33 (0.47)	48.16 (0.69)	50.12 (2.17)	**0.002**

a CNMI= Commonwealth of the Northern Mariana Islands.

**Table 3:** Daily Average Intake of Food Groups, Macronutrients, and Micronutrients of Filipino Children by Pacific Jurisdiction, adjusted for age, sex, and energy intake.

Dietary Intake	Total	CNMI^a^	Guam	Hawai⊠i	P-value
	Mean (SE)	Mean (SE^b^)	Mean (SE)	Mean (SE)	
**FOOD GROUPS**					
Grains (CETK^c^)	6.47 (0.05)	6.52 (0.07)	6.42 (0.08)	6.39 (0.08)	0.37
Fruits (CETK)	0.95 (0.12)	0.97 (0.12)	0.85 (0.11)	1.00 (0.15)	0.46
Vegetables (CETK)	0.64 (0.05)	0.66 (0.04)	0.61 (0.04)	0.65 (0.04)	0.30
Dairy ( CETK)	1.39 (0.13)	1.27 (0.13)	1.39 (0.13)	1.55 (0.14)	**0.04**
Meat (CETK)	5.64 (0.25)	6.26 (0.19)	5.24 (0.07)	5.00 (0.34)	**0.002**
MACRONUTRIENTS					
Total energy (kcal)	1703.27 (15.95)	1715.65 (29.67)	1718.70 (30.80)	1660.56 (24.22)	0.38
Total protein (g)	4.26 (0.98)	4.20 (0.06)	4.55 (0.07)	4.02 (0.25)	**0.0004**
Total carbohydrates (g)	221.96 (1.36)	220.24 (0.81)	221.65 (0.94)	224.55 (4.34)	0.41
Total Fiber (g)	9.54 (0.29)	9.44 (0.35)	8.64 (0.24)	10.32 (0.62)	0.05
Total fat(g)	62.27 (0.52)	61.35 (0.32)	63.64 (0.29)	62.67 (1.09)	**0.0006**
Linolenic Acid (g)	1.19 (0.03)	1.19 (0.01)	1.24 (0)	1.17 (0.02)	**0.004**
Linoleic Acid (g)	10.70 (0.15)	10.45 (0.08)	11.43 (0.07)	10.58 (0.12)	**< .0001**
Saturated fat (g)	21.71 (0.30)	20.96 (0.08)	22.05 (0.25)	22.59 (0.39)	**0.002**
Trans Fat (g)	0.62 (0.04)	0.51 (0.04)	0.63 (0.05)	0.77 (0.06)	**0.002**
Cholesterol (mg)	258.82 (8.07)	280.0 (4.17)	244.58 (5.77)	237.22 (9.85)	**0.001**
MICRONUTRIENTS - VITAMINS					
Choline (mg)	266.83 (6.03)	282.41 (4.95)	252.83 (3.52)	253.88 (5.86)	**0.001**
Folate (μg DFE)	424.67 (11.54)	410.27 (10.42)	470.62 (8.36)	416.04 (20.61)	**0.002**
Niacin (mg)	18.31 (0.32)	18.81 (0.51)	18.65 (0.23)	17.41 (0.28)	**0.02**
Pantothenic Acid (mg)	4.60 (0.13)	4.85 (0.05)	4.45 (0.03)	4.32 (0.05)	**< .0001**
Riboflavin (mg)	1.75 (0.11)	1.72 (0.13)	1.73 (0.13)	1.79 (0.13)	0.44
Thiamin (mg)	1.29 (0.05)	1.28 (0.05)	1.32 (0.05)	1.28 (0.07)	0.55
Vitamin A (μg RAE)	499.58 (10.49)	503.83 (11.95)	487.58 (23.18)	503.13 (29.08)	0.83
Vitamin B-6 (mg)	1.50 (0.06)	1.58 (0.08)	1.45 (0.06)	1.41 (0.06)	0.09
Vitamin B-12 (mg)	4.95 (0.13)	5.07 (0.19)	4.90 (0.18)	4.86 (0.18)	0.23
Vitamin C (mg)	67.99 (1.98)	66.72 (2.32)	70.66 (4.56)	68.91 (5.09)	0.72
Vitamin D (IU)	204.41 (3.26)	199.53 (3.75)	209.94 (5.93)	208.08 (7.5)	0.32
Vitamin E (mg)	4.46 (0.04)	4.42 (0.03)	4.69 (0.02)	4.39 (0.04)	**< .0001**
MICRONUTRIENTS - MINERALS					
Calcium (mg)	689.29 (18.86)	641.67 (14.11)	682.64 (14.53)	763.27 (30.82)	**0.02**
Copper (mcg)	0.92 (0.03)	0.94 (0.04)	0.91 (0.04)	0.92 (0.04)	0.43
Iron (mg)	12.09 (0.18)	12.22 (0.31)	12.15 (0.08)	11.91 (0.40)	0.81
Magnesium (mg)	208.3 (4.01)	215.07 (4.65)	196.86 (1.75)	206.13 (5.83)	**0.005**
Manganese (mg)	2.51 (0.10)	2.58 (0.07)	2.41 (0.07)	2.48 (0.08)	**0.007**
Phosphorous (mg)	1012.28 (11.15)	1018.58 (11.16)	966.47 (14.23)	1033.5 (23.16)	**0.03**
Selenium (μg)	100.92 (1.95)	106.07 (0.78)	97.59 (0.84)	95.36 (1.38)	**< .0001**
Sodium (mg)	2473.36 (38.44)	2436.2 (54.28)	2452.09 (25.53)	2547.41 (62.17)	0.36
Potassium (mg)	1938.49 (37.76)	1999.48 (52.1)	1847.71 (19.9)	1916.3 (59.42)	**0.03**
Zinc (mg)	9.17 (0.08)	9.13 (0.11)	9.05 (0.19)	9.31 (0.16)	0.60
OTHER FOOD COMPOUNDS					
Nitrate (mg)	40.14 (0.88)	40.55 (1.17)	37.35 (0.74)	41.74 (1.70)	0.07
Nitrite (mg)	1.27 (0.10)	1.36 (0.10)	1.25 (0.10)	1.14 (0.11)	**0.002**

a CNMI= Commonwealth of the Northern Mariana Islands.

b SE=Standard Error

c CETK=cup equivalents per 1,000 kcal consumed

d DFE= Dietary Folate Equivalents

e RAE= Retinol Activity Equivalents

## Data Availability

Data is available upon request to the corresponding author.
